# Integrated spectral photocurrent density and reproducibility analyses of excitonic ZnO/NiO heterojunction

**DOI:** 10.1016/j.dib.2017.09.007

**Published:** 2017-09-12

**Authors:** Malkeshkumar Patel, Joondong Kim

**Affiliations:** a Department of Electrical Engineering, Incheon National University, 119 Academy Rd. Yeonsu, Incheon 406772, Republic of Korea; b Photoelectric and Energy Device Application Lab (PEDAL), Multidisciplinary Core Institute for Future Energies (MCIFE), Incheon National University, 119 Academy Rd. Yeonsu, Incheon 406772, Republic of Korea

**Keywords:** Metal oxides, ZnO/NiO transparent, Solar cells, Stability, Phase structure

## Abstract

In this data article, the excitonic ZnO/NiO heterojunction device (Patel et al., 2017) [1] was measured for the integrated photocurrent density and reproducibility. Photograph of the prepared devices of ZnO/NiO on the FTO/glass is presented. Integrated photocurrent density as a function of photon energy from the sunlight is presented. Quantum efficiency measurement system (McScienceK3100, Korea) compliance with International Measurement System was employed to measure ZnO/NIO devices. These data are shown for the 300–440 nm of segment of the sunlight (AM1.5G, http://rredc.nrel.gov/solar/spectra/am1.5/). Reproducibility measure of ZnO/NiO device was presented for nine devices with the estimated device performance parameters including the open circuit voltage, short circuit current density, fill factor and power conversion efficiency.

**Specifications Table**

**Table:** 

Subject area	*Physics, Electrical Engineering*
More specific subject area	*Solar cells*
Type of data	*Figures, Table*
How data was acquired	*Quantum efficiency measurement system (McScienceK3100, Korea)*
	*Potentiostat/Galvanostat (ZIVE SP1, WonA Tech, Korea)*
Data format	*Analyzed*
Experimental factors	*J-V: Linear sweep voltammetry, positive direction, scan range 0*–*0.8* *V, compliance auto, scan resolution 5* *mV.*
	*Spectral photoresponse: reference cell-Si photodiode, scan range 300*–*450* *nm, room temperature.*
Experimental features	*Excitonic metal oxide heterojunction (NiO/ZnO) solar cells*
Data source location	*Incheon National University, Incheon-406772, Korea*
Data accessibility	*The data are with this article*

**Value of the data**

• Photograph of the prepared ZnO/NiO devices for the transparent feature and reproducibility of the fabrication.
• Integrated photocurrent density of ZnO/NiO device for UV light would be useful to design UV operational transparent solar cells.
• Reproducibility and statistical information of the excitonic ZnO/NiO/Ag devices would be useful to demonstrate consistency.

## 1 Data

**Fig. 1:**
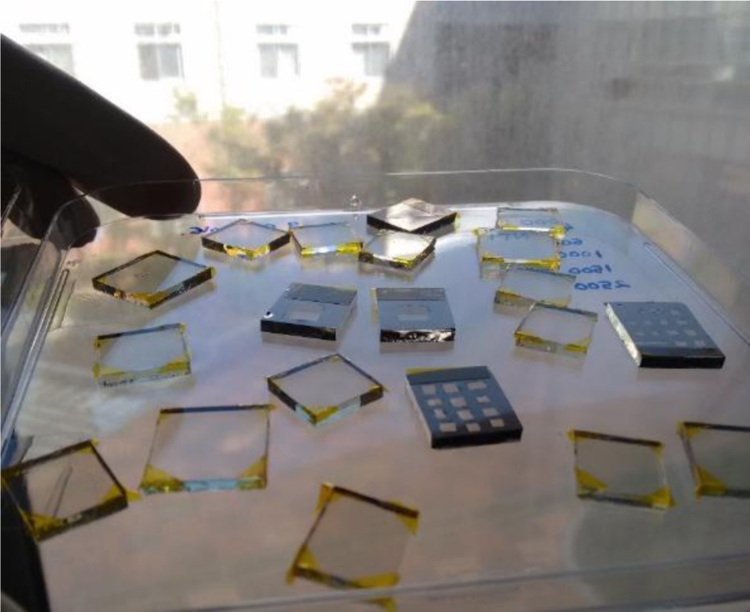
Prepared devices to study the reproducibility of ZnO/NiO structure on the FTO/glass substrate.

**Fig. 2:**
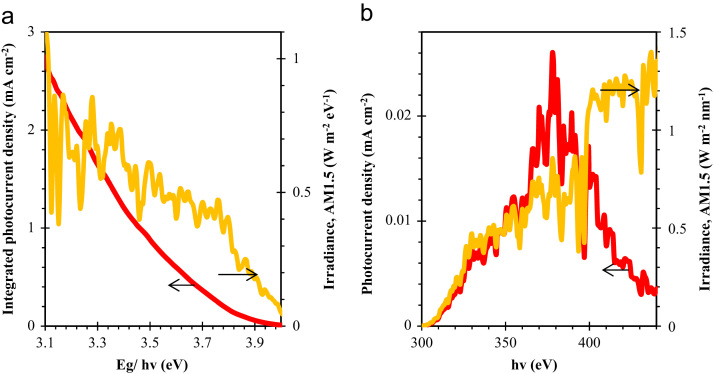
(a) Integrated photocurrent density as a function of photon energy from the sunlight and irradiance spectral distribution. Spectral distribution of AM1.5G (http://rredc.nrel.gov/solar/spectra/am1.5/) was processed for photocurrent density. Each photon absorption generate one electron/hole pair was considered. (b) Photocurrent density of ZnO/NiO device estimated using the quantum efficiency data as shown in Fig. 3b in our manuscript.

**Fig. 3:**
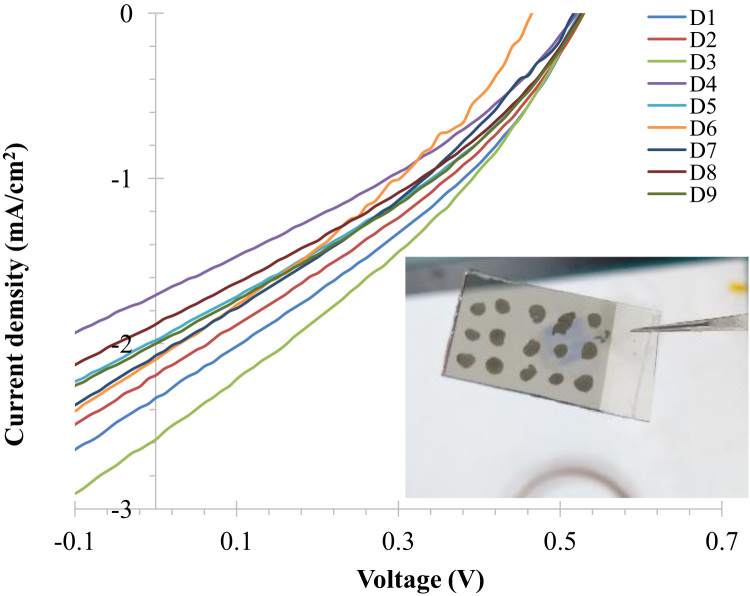
Reproducibility measure of excitonic ZnO/NiO/Ag device, *J*–*V* characteristics under the UV light (365 nm, 8 mW/cm^2^). Inset shows the photograph of the prepared sample.

Fig. 1 shows the devices of ZnO/NiO prepared on the FTO/glass substrate to study the reproducibility and stability. Integrated photocurrent density as a function of photon energy from the sunlight and irradiance spectral distribution AM1.5G (available online in Ref. [2]) was processed for photocurrent density and are shown in Fig. 2 after considering that each photon absorption in ZnO layer generates one electron/hole pair. Reproducibility measure of ZnO/NiO device was performed for the Ag paste applied total nine devices. These devices were measured for J-V characteristics as presented in Fig. 3. Estimated power conversion parameters such as open circuit voltage, short circuit current density, fill factor and power conversion efficiency of these devices are summarized in Table 1.

**Table 1:** Estimated solar cell performances. The native *J*–*V* spectra are shown in the Fig. 3. *V*_OC_, *J*_SC_, FF and Efficiency are summarized.

**Device**	***V***_**OC**_**(V)**	***J***_**SC**_**(mA/cm**^**2**^**)**	**FF (%)**	**Effi (%)**
D1	0.526	2.33±0.058	32.76	5.02
D2	0.526	2.19±0.054	32.33	4.65
D3	0.526	2.57±0.64	32.29	5.46
D4	0.516	1.70±0.042	33.17	3.64
D5	0.526	1.97±0.042	32.98	4.29
D6	0.466	2.09±0.052	31.71	3.86
D7	0.516	2.07±0.051	28.91	3.86
D8	0.526	1.856±0.046	33.69	4.11
D9	0.526	2.00±0.050	33.22	4.37
Average	0.516	1.971	32.30	4.10

## 2 Experimental design, materials and methods

### 2.1 Sample preparation

Glass and commercial FTO glass were used as substrates and was cleaned according to Ref. [1]. ZnO film was formed using the RF magnetron sputtering. Conditions for preparing ZnO sample is as follows.

**Table:** 

Target	ZnO (Ø4 inch, purity 99.999%)
RF power	300 W
Gas/flow rate	Ag/50 sccm
Deposition pressure	5 mTorr
Temperature	Room temperature
Substrate rotation	5 rpm
Deposition time	30 minutes

NiO film was formed using the DC reactive sputtering. Conditions for preparing NiO sample are as follows.

**Table:** 

Target	Ni (Ø4 inch, purity 99.999%)
DC power	50 W
Gas/flow rate	Ag/50 sccm, O_2_/4 sccm
Deposition pressure	3 mTorr
Temperature	Room temperature
Substrate rotation	5 rpm
Deposition time	15 minutes

For the transparent solar cell, FTO-coated glass was used. The deposition condition of UV-reactive layers (ZnO and NiO) was same as above. Ag paste was applied above the NiO layer as conducting electrode.

### 2.2 Sample characterizations

Integrated photocurrent density as a function of photon energy (hv) from the Sunlight as irradiance spectral distribution applied. AM 1.5G data were applied for this calculation and available on Ref. [2]. For this calculation, we considered that each-photon absorption in ZnO/NiO device generates one electron/hole pair and this data are shown in Fig. 2. The measured quantum efficiency of ZnO/NiO/Ag paste was applied to estimate the photocurrent density from the sunlight. The normalized quantum efficiency data are presented in the Fig. 3b of the Ref. [1]. This measurement was performed using a quantum efficiency measurement system (McScienceK3100, Korea) after the calibrating using reference Si photodiode in the scan range 300 nm to 450 nm at room temperature.

*J*–*V* characteristics of ZnO/NiO/Ag devices are presented in Fig. 3. These data were measured for UV illumination. Illuminated *J*–*V* spectra were applied to estimate the solar cell performance parameters such as open circuit voltage (*V*_OC_), short circuit current density (*J*_SC_), fill factor (FF), and power conversion efficiency which are summarized in Table 1.
