# Supplementary material for: Integrated spectral photocurrent density and reproducibility analyses of excitonic ZnO/NiO heterojunction
Source: Data Brief. 2017 Sep 12;15:81–5. doi: 10.1016/j.dib.2017.09.007 (PMC5614732; doi:10.1016/j.dib.2017.09.007)
Supplement: Supplementary file 1 — Transparency document [file mmc1.docx]

***Conflicts of Interest Statement***

Re: DIB-D-17-00551R2

Title: *Phase structure and stability analyses of excitonic ZnO/NiO transparent solar cells*

We declare that this manuscript is original, has not been reported before, and is not currently being considered elsewhere. We also confirm that there is no known conflict of interest regarding this manuscript and its publication. The manuscript has been approved by all named authors.

Sincerely yours,


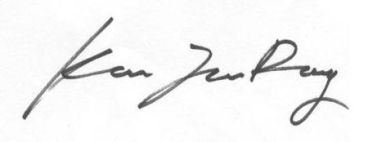


Joondong Kim

Joondong Kim, Ph.D./Professor

Department of Electrical Engineering,

Incheon National University

E-mail: joonkim@ incheon.ac.kr

Phone: +82-32-835-8770; fax: +82-32-835-0773
